# Application of the International System for Reporting Serous Fluid Cytopathology (ISRSFC) in reporting serous effusion: A retrospective study

**DOI:** 10.1097/MD.0000000000035707

**Published:** 2023-10-27

**Authors:** Haiping Yang, Jianyou Zhu, Pingjiang Wang

**Affiliations:** a Departments of Pathology, Linzi District People’s Hospital, Zibo, China; b Departments of Gastrointestinal Surgery, Linzi District People’s Hospital, Zibo, China.

**Keywords:** ISRSFC, performance analysis, risk of malignancy, serous effusion

## Abstract

In order to develop uniform diagnostic standards and reporting terminology, the International Academy of Cytology and the American Society of Cytopathology have recommended the establishment of the International System for Reporting Serous Fluid Cytopathology (ISRSFC). ISRSFC has 5 diagnostic categories: non-diagnostic (ND), negative for malignancy (NFM), atypia of unknown significance (AUS), suspicious for malignancy (SFM), and malignant (MAL). So far, very few studies have evaluated the risk of malignancy (ROM) and performance characteristics (sensitivity, specificity, positive predictive value, negative predictive value and diagnostic accuracy) of different categories. The purpose of this study was to reclassify serous effusions based on the ISRSFC and to assess their ROM and performance characteristics. All serous effusions from January 2017 to December 2022 were categorized according to the ISRSFC. Using histopathological diagnosis as the gold standard, the ROM and performance characteristics were calculated for each group. Finally, a total of 2103 serous effusion specimens were analyzed. After reclassification, 9 (0.4%) cases were classified as ND, 547 (26%) as NFM, 94 (4.5%) as AUS, 386 (18.4%) as SFM, and 1067 (50.7%) as MAL. The ROMs for ND, NFM, AUS, SFM and MAL were calculated to be 50%, 24.9%, 36.8%, 89.0%, and 100%, respectively. As an easy-to-grasp reporting system, ISRSFC provides a consistent standard for better communication between physicians and pathologists.

## 1. Introduction

Fluid buildup inside the serous cavity often occurs in neoplastic and non-neoplastic conditions, such as inflammation, infection, organ failure, injury, and malignancy.^[1]^ Since serous effusion tends to be the first clinical manifestation of patients, coupled with being easily accessible, less invasive, and low cost, serous cytological examination has been widely used by clinicians to differentiate between benign and malignant effusion and to investigate the cause of the effusion.^[2,3]^ Furthermore, in patients with malignant tumors, serous cytology can determine whether the tumor is primary or secondary, and in the latter instance, identify the primary site when effusion presents as the first symptom. It also aids in the staging of malignant tumors, recurrence, prognosis evaluation, and formulation of treatment strategy. Therefore, it is essential to perform precise pathological examinations of effusion specimens from cancer patients.^[4,5]^ Despite its widespread use, there is no definitive method for reporting effusion cytology. Diagnostic terminology reported by different laboratories varies considerably when lymphocytes, reactive mesothelial cells, and histiocytes predominate, or the cellular appearance of benign and malignant entities overlaps. As a result, the International Academy of Cytology and the American Society of Cytopathology supported a team of cytology specialists to publish “The International System for Reporting Serous Fluid Cytopathology” (ISRSFC) to provide uniform diagnostic guidelines and reporting nomenclature.^[6–8]^ This evidence-based reporting system was designed to enhance communication between physicians and cytopathologists and to guide them in improving patient management and treatment choices.

The ISRSFC has 5 diagnostic categories: nondiagnostic (ND), negative for malignancy (NFM), atypia of unknown significance (AUS), suspicious for malignancy (SFM), and malignant (MAL, primary or secondary). The AUS and SFM groups are considered to have an indeterminate diagnosis, which can be clinically challenging to manage and may prompt further diagnostic tests, increasing patient expenditures and morbidity.

In this study, this novel categorization system was tested and its diagnostic criteria were applied to peritoneal, pleural and pericardial effusion samples. After specimen reclassification, the risk of malignancy (ROM) in each group was computed, and sensitivity, specificity, positive predictive value (PPV), negative predictive value (NPV), and diagnostic accuracy were evaluated.

## 2. Materials and methods

### 2.1. Data collection and specimens preparation

The Ethics Committee of Linzi District People’s Hospital approved this retrospective study on the application of ISRSFC in pleural, peritoneal, and pericardial effusion specimens. All the serous effusion samples were collected between January 2017 and December 2022. Peritoneal washing was not performed in this study. Data were gathered on patient demographics, clinical symptoms, cytological and histological reports, IHC results of the cell block, and patient treatment. All patients were reclassified separately by 2 cytopathologists according to the ISRSFC criteria. If there was some disagreement between the 2 cytopathologists in some cases, the same diagnosis was made after discussion.

Fresh specimens were collected from the anticoagulant-free containers. All the samples were processed within 4 hours. The material was centrifuged for 10 minutes at 2000 revolutions per minute, and the supernatant was discarded. The cell sediment was separated into 2 portions to prepare conventional cell smears and formalin-fixed paraffin-embedded cell blocks. All cell smears and cell blocks were stained with hematoxylin and eosin. Cell blocks were routinely prepared for all samples unless there was insufficient material. Immunohistochemistry was performed on cell blocks to confirm the tumor and to further identify the type and probable primary site when malignancy was suspected. There are various immunohistochemical markers, including Wilms’ tumor 1, Calretinin, P63, cytokeratin 5/6, cytokeratin 7 (CK7), cytokeratin 20, thyroidtranscription factor-1, Napsin A, cancer antigen 125, caudal type homeobox 2, GATA- binding protein 3, mammaglobin, estrogen receptor, progesterone receptor, and human epidermal growth factor receptor 2.

### 2.2. Reclassification

**Figure 1.**
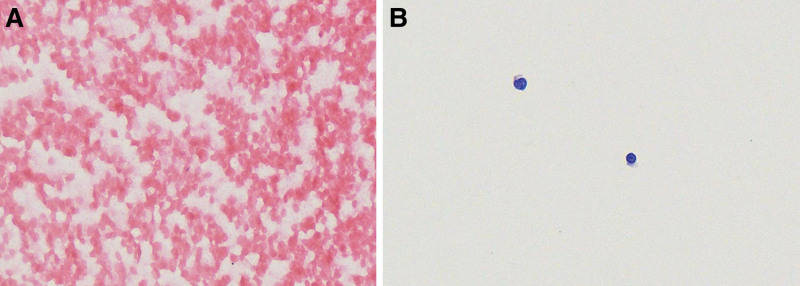
Non-diagnostic. (A) Cytosmear showing no cells, only blood (HE, 400×). (B) Cytosmear showing extremely few cells (<10) (HE, 400×).

The features of each specimen, including volume, microscopic descriptions of cytosmear slides, cell blocks, and IHC slides were analyzed by 2 experienced cytopathologists separately and blindly. Based on the gathered data, all cases were reclassified using the criteria outlined in the ISRSFC. In summary, they are as follows:

*Non-diagnostic*: Hemorrhagic specimens or specimens with no or insufficient cells (fewer than 10 cells), such as mesothelial cells, macrophages, or lymphocytes (Fig. 1).

**Figure 2.**
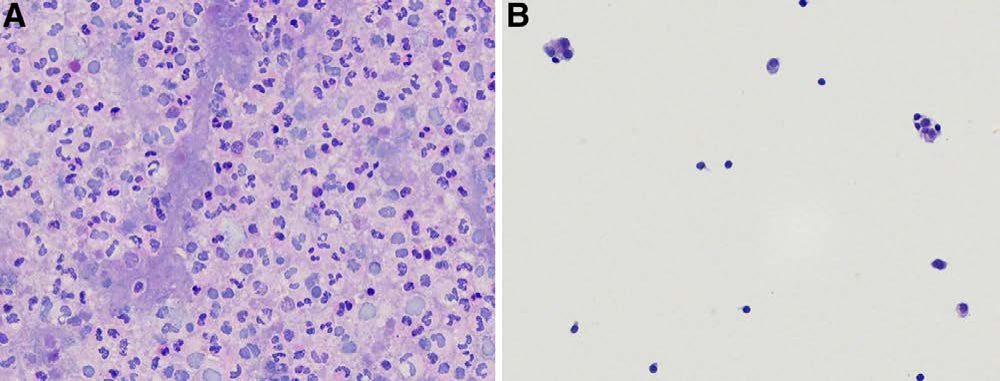
Negative for malignancy. (A) Cytosmear showing sheets of neutrophils and a few macrophages (HE, 400×). (B) Cytosmear showing an abundance of cells, including reactive mesothelial cells and lymphocytes (HE, 400×).

*Negative for malignancy*: The sample consisted of one or more cell types without cell atypia or signs of malignancy. The morphology of mesothelial cells, macrophages, and lymphocytes is benign, regardless of the clinical history or imaging features (Fig. 2).

**Figure 3.**
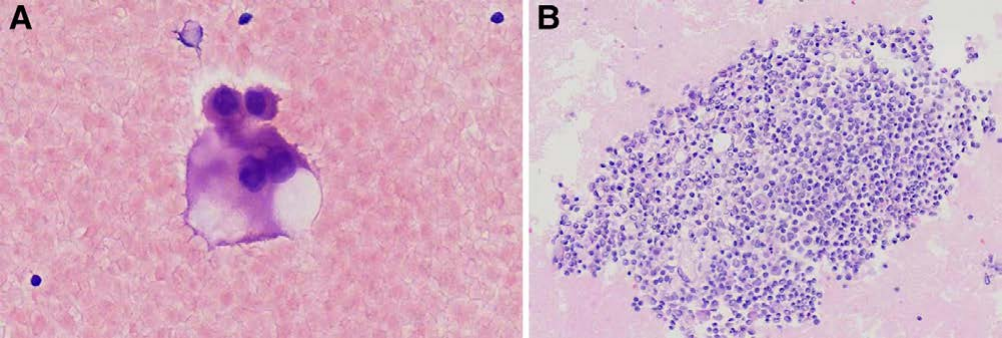
Atypia of unknown significance. (A) A cluster of atypical epithelioid cells with a high nuclear-to-cytoplasmic ratio and irregular nuclear membranes can be seen on the cytosmear (HE, 400×). (B) Section from cell block showing reactive mesothelial cells, macrophages, and lymphocytes (HE, 400×).

*Atypia of unknown significance*: Samples consisted of atypical cells but lacking quantitative or qualitative cytological characteristics to confirm a benign or malignant diagnosis. Atypical morphological manifestations are less likely to be malignant and are more likely to be reactive or degenerative. Most of the AUS cases tended to be benign (Fig. 3).

**Figure 4.**
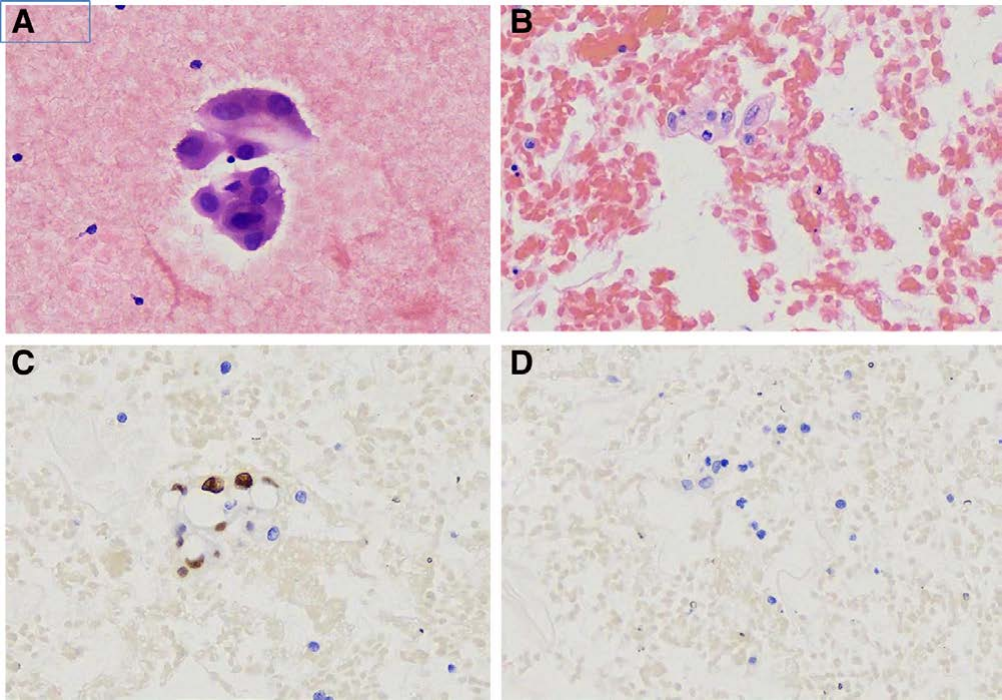
Suspicious for malignancy. (A) Atypical epithelioid cells with a high nuclear to cytoplasmic ratio in a patient with a history of lung cancer (HE, 400×). (B) Section from cell block showing similar atypical cells (HE, 400×). These atypical cells were TTF-1 (C, immunohistochemical staining, 400×) immunopositive but Calretinin (D, immunohistochemical staining, 400×) negative. TTF-1 = thyroidtranscription factor-1.

*Suspicious for malignancy*: The samples contained significant abnormal cells (usually found in malignant tumors), but not in sufficient numbers for a definitive diagnosis of malignancy. The diagnosis of SFM tended to be malignant (Fig. 4).

**Figure 5.**
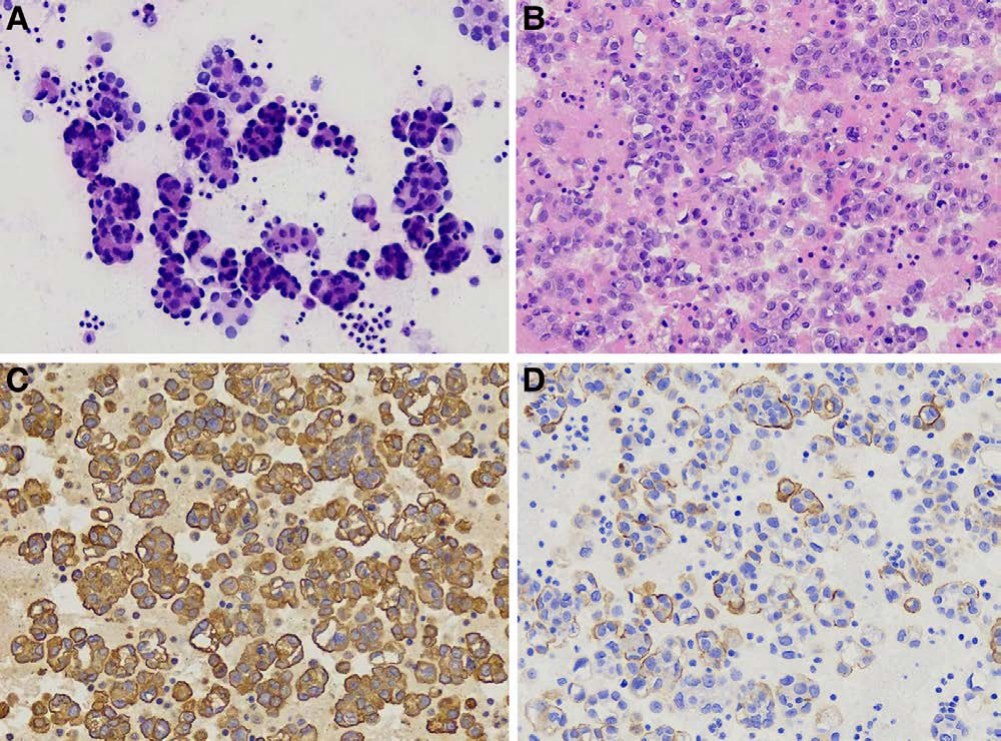
Malignancy. (A) Cytosmear revealing clusters of papillary cells with significant atypia and prominent, hyperchromatic nuclei (HE, 400×). (B) Section from cell block demonstrating significant cellularity with malignant papillary cells (HE, 400×). These tumor cells were immunopositive for EMA (C, immunohistochemical staining, 400×) and CA125 (D, immunohistochemical staining, 400×).

*Malignancy*: Samples exhibiting malignant characteristics, either alone or in combination with the results of ancillary techniques, are sufficient to diagnose a primary or secondary malignancy (Fig. 5).

### 2.3. Statistical analysis

Data were summarized in an Office Excel spreadsheet, with each row representing a cytological specimen. Histological diagnosis, that is biopsy or postoperative pathological findings, is regarded as the gold standard for calculating the ROM and performance analysis.

Performance analysis included calculation of sensitivity, specificity, PPV, NPV, and diagnostic accuracy for peritoneal, pleural, and pericardial effusion samples. Performance analysis calculations were performed separately based on MAL as the positive group, MAL and SFM as the positive groups, and MAL, SFM, and AUS as the positive groups. Patients in the ND group were excluded from the performance analysis because they could be categorized as positive or negative for malignancy. The following formulas^[9,10]^ were used to compute ROM, sensitivity, specificity, PPV, NPV, and diagnostic accuracy:

ROM: Number of cases with a final diagnosis of malignancy × 100%/total number of cases

Sensitivity: True positive × 100%/(True positive + False negative)

Specificity: True negative × 100%/(True negative + False positive)

PPV: True positive × 100%/(True positive + False positive)

NPV: True negative × 100%/(True negative + False negative)

Diagnostic accuracy: (True positive + True negative) × 100%/All analyzed cases

## 3. Results

### 3.1. Diagnosis before reclassification

Before reclassification, cytopathic effusion samples were diagnosed based on cell morphology and morphology on the cell block, as well as immunohistochemical findings on the cell blocks. In our institution, the initial diagnosis was highly different, and the terminology used was changed from pathologist to pathologist. Despite the lack of rigorous criteria, 21 (1%) cases with only blood or a handful of cells were considered “undiagnosable.” A total of 629 (29.9%) cases described as “benign effusion,” “negative effusion,” or “no malignant cells” by different cytopathologists were grouped as “benign.” This diagnosis was made when the cytosmears demonstrated only reactive mesothelial cells, macrophages, and lymphocytes. Another 393 (18.7%) patients were diagnosed with “suspected malignancy,” “presence of atypical cells,” or “uncertain malignancy,” and were lumped together as “suspicious of malignancy.” This diagnosis was made when the cytosmears revealed atypical cells, and it was not possible to differentiate between a benign reactive process and a malignant tumor. The remaining 1060 (50.4%) cases were diagnosed as malignant because the cytosmears showed a high density of cells with definite malignant cells, which were reported as “positive effusion,” “presence of malignant cells,” “metastatic cancer cells,” and “malignant cells found.” The initial diagnoses for each case are summarized in Table 1.

**Table 1:** Distribution of cases according to the initial cytodiagnosis and after recategorization.

Initial diagnostic categories	N (%)	ISRSFC categories	N (%)
Undiagnosable	21 (1%)	ND	9 (0.4%)
Benign	629 (29.9%)	NFM	547 (26%)
Suspicious of malignancy	393 (18.7%)	AUS	94 (4.5%)
		SFM	386 (18.4%)
Malignancy	1060 (50.4%)	MAL	1067 (50.7%)
Total	2103		2103

AUS = atypia of unknown significance, ISRSFC = International System for Reporting Serous Fluid Cytopathology, MAL = malignancy, ND = non-diagnostic, NFM = negative for malignancy, SFM = suspicious of malignancy.

### 3.2. Patient demography

Between January 2017 and December 2022, 2103 serous effusion specimens were enrolled in the study, of which 1199 (57.0%), 790 (37.6%), and 114 (5.4%) were pleural, peritoneal, and pericardial effusions, respectively. The patients ranged in age from 16 to 102 years, with an average age of 67.1 years. The patients were primarily men (n = 1180), with a male-to-female ratio of 1.3:1. The volume of the serous effusions ranged from 5 to 1000 mL (mean: 186 mL). Cell blocks were produced in 1342 cases, of which 508 were subjected to immunohistochemistry. After reclassification, 9 (0.4%) cases were classified as ND, 547 (26%) as NFM, 94(4.5%) as AUS, 386 (18.4%) as SFM, and 1067 (50.7%) as MAL. A total of 776 samples were excluded from ROM analysis because there were no matched biopsies or postoperative pathology data. Finally, 667 pleural effusions, 582 peritoneal effusions, and 78 pericardial effusions were used to calculate ROM for each group. The demographic and specimen characteristics of the patients and the ROMs calculated for each group are shown in Tables 2 and 3, respectively.

**Table 2:** Patient demographics and specimen features of 2103 serous effusions based on each ISRSFC category.

Diagnostic category	ND	NFM	AUS	SFM	MAL	Total
Number of patients, n (%)	9 (0.4)	547 (26)	94 (4.5)	386 (18.4)	1067 (50.7)	2103
Gender (number of men/women)	6/3	361/186	50/44	271/115	492/575	1180/923
Age range	42–74	16–102	24–90	40–79	47–92	16–102
Serous effusion source (n)						
Pleural	5	317	56	242	579	1199
Peritoneal	2	189	26	144	429	790
Pericardial	2	41	12	0	59	114
Cell block slides (n)	4	323	52	385	578	1342
IHC (n)	0	197	24	101	186	508

AUS = atypia of unknown significance, IHC = immunohistochemistry, ISRSFC = International System for Reporting Serous Fluid Cytopathology, MAL = malignancy, ND = non-diagnostic, NFM = negative for malignancy, SFM = suspicious of malignancy.

**Table 3:** The risk of malignancy in our study based on each ISRSFC category.

Specimen type	Total cases	ROM				
		ND	NFM	AUS	SFM	MAL
Pleural effusion	667	50% (1/2)	31.0% (75/242)	37.0% (10/27)	91.8% (67/73)	100% (323/323)
Peritoneal effusion	582	0 (0/1)	21.0% (56/267)	36.8% (7/19)	85.2% (69/81)	100% (214/214)
Pericardial effusion	78	100% (1/1)	8.0% (2/25)	36.4% (4/11)	100% (9/9)	100% (32/32)
Total	1327	50% (2/4)	24.9% (133/534)	36.8% (21/57)	89.0% (145/163)	100% (569/569)

AUS = atypia of unknown significance, ISRSFC = International System for Reporting Serous Fluid Cytopathology, MAL = malignancy, ND = non-diagnostic, NFM = negative for malignancy, ROM = risk of malignancy, SFM = suspicious of malignancy

### 3.3. The ROM and performance analysis in pleural, peritoneal, and pericardial effusion specimens

#### 3.3.1. Pleural effusions

According to ISRSFC standards, 667 pleural effusions were categorized as follows: 2 (0.3%) ND, 242 (36.3%) NFM, 27 (4.1%) AUS, 73 (10.9%) SFM, and 323 (48.4%) MAL. The ROM for ND was 50% (1/2), for NFM was 31.0% (75/242), for AUS was 37.0% (10/27), for SFM was 91.8% (67/73), and for MAL was 100% (323/323). When only the MAL category was considered as the positive group, there were 152 false-negative cases and no false-positive cases. The sensitivity, specificity, PPV, NPV, and diagnostic accuracy were 68%, 100%, 100%, 55.8%, and 77.2%, respectively. There were 85 false-negative cases and 6 false-positive cases in the MAL and SFM categories as positive groups. Sensitivity, specificity, PPV, NPV, and diagnostic accuracy were 82.3%, 96.8%, 98.5%, 67.9%, and 86.4%, respectively. Considering the MAL, SFM, and AUS categories as positive groups, there were 75 false-negative and 23 false-positive cases. Sensitivity, specificity, PPV, NPV, and diagnostic accuracy were 84.9%, 86.4%, 94.8%, 66.0%, and 85.3%, respectively.

The most common malignancy in pleural effusion cases was lung adenocarcinoma (63.2%), followed by breast carcinoma (8.6%), gastric carcinoma (7.8%), liver carcinoma (5.3%), esophageal carcinoma (4.4%), colorectal carcinoma (4.2%), ovarian carcinoma (3.4%), pancreatic carcinoma (2.1%), thymic carcinoma (0.6%), and mesothelioma (0.4%). Ancillary immunohistochemistry was performed on 377 pleural effusion specimens. Immunohistochemistry results for lung adenocarcinoma are shown in Figure 4.

#### 3.3.2. Peritoneal effusions

A total of 582 peritoneal effusions were reclassified using the ISRSFC criteria as follows: 1 (0.17%) ND, 267 (45.9%) NFM, 19 (3.3%) AUS, 81 (13.9%) SFM, and 214 (36.8%) MAL. The ROM was 0 (0/1) for ND, 21.0% (56/267) for NFM, 36.8% (7/19) for AUS, 85.2% (69/81) for SFM, and 100% (214/214) for MAL. When only the MAL category was considered as the positive group, there were 132 false-negative cases and no false-positive cases. The sensitivity, specificity, PPV, NPV, and diagnostic accuracy were 61.8%, 100%, 100%, 64.1%, and 77.3%, respectively. When considering the MAL and SFM categories as positive groups, there were 63 false-negative and 12 false-positive cases. Sensitivity, specificity, PPV, NPV, and diagnostic accuracy were 82.4%, 94.6%, 96.1%, 77.1%, and 87.1%, respectively. Considering the MAL, SFM, and AUS categories as positive groups, there were 56 false-negative and 24 false-positive cases. Sensitivity, specificity, PPV, NPV, and diagnostic accuracy were 84.9%, 88.7%, 92.9%, 77.0%, and 86.3%, respectively.

Gastrointestinal carcinoma (56.8%) was the most common malignancy, followed by hepatobiliary carcinoma (22.4%), ovarian carcinoma (14.7%), lung carcinoma (5.0%), breast carcinoma (0.73%), and lymphoma (0.37%). Ancillary immunohistochemistry was performed on 102 specimens of peritoneal effusion. Immunohistochemistry results for ovarian cancer are shown in Figure 5.

#### 3.3.3. Pericardial effusions

According to ISRSFC standards, 78 pericardial effusions were reclassified as follows: 1 (1.3%) ND, 25 (32.1%) NFM, 11 (14.1%) AUS, 9 (11.5%) SFM, and 32 (41.0%) MAL. The ROM was 100% (1/1) for ND, 8.0% (2/25) for NFM, 36.4% (4/11) for AUS, 100% (9/9) for SFM, and 100% (32/32) for MAL. When only the MAL category was considered as the positive group, there were 15 false-negative cases and no false-positive cases. Sensitivity, specificity, PPV, NPV, and diagnostic accuracy were 68.0%, 100%, 100%, 67.4%, and 80.8%, respectively. When considering the MAL and SFM categories as positive groups, there were 6 false-negative cases and no false-positive cases. Sensitivity, specificity, PPV, NPV, and diagnostic accuracy were 87.2%, 100%, 100%, 83.8%, and 56.9%, respectively. When considering the MAL, SFM, and AUS categories as positive groups, there were 2 false-negative cases and 7 false-positive cases. Sensitivity, specificity, PPV, NPV, and diagnostic accuracy were 96.3%, 70.8%, 88.1%, 89.5%, and 88.5%, respectively.

The most common malignancy detected in patients with pericardial effusion was lung adenocarcinoma (89.9%), followed by gastric carcinoma (4.4%), renal carcinoma (2.9%), pancreatic carcinoma (1.4%), and esophageal carcinoma (1.4%). Twenty-nine pericardial effusion specimens were subjected to ancillary immunohistochemistry (IHC). The performance analysis of pleural, peritoneal, and pericardial effusion specimens is shown in Table 4.

**Table 4:** The performance analysis among different serous effusions.

Specimen type		MAL + SFM + AUS positive (%)	MAL + SFM positive (%)	MAL positive (%)
Pleural effusion	Sensitivity	84.9	82.3	68
	Specificity	86.4	96.8	100
	PPV	94.8	98.5	100
	NPV	66.0	67.9	55.8
	Diagnostic accuracy	85.3	86.4	77.2
Peritoneal effusion	Sensitivity	84.9	82.4	61.8
	Specificity	88.7	94.6	100
	PPV	92.9	96.1	100
	NPV	77.0	77.1	64.1
	Diagnostic accuracy	86.3	87.1	77.3
Pericardial effusion	Sensitivity	96.3	87.2	68.0
	Specificity	70.8	100	100
	PPV	88.1	100	100
	NPV	89.5	83.8	67.4
	Diagnostic accuracy	88.5	56.9	80.8

AUS = atypia of unknown significance, MAL = malignancy, NPV = negative predictive value, PPV = positive predictive value, SFM = suspicious of malignancy.

## 4. Discussion

Serous cytology is a very useful tool for detecting malignant tumors and guiding treatment options in clinical practice. Hence, the correct diagnosis of effusion is critical for the medical treatment of patients. Serous effusions were previously classified as benign or malignant with inconsistent diagnostic criteria and reporting terms. In order to address the issues mentioned above and better guide clinical therapy, the International Academy of Cytology and the American Society of Cytopathology produced “ISRSFC.” In the current study, the suitability of this novel approach for reporting cytopathological results of pleural, peritoneal, and pericardial effusions was evaluated.

Before reclassification, the initial diagnostic classifications were broadly divided into 4 groups: undiagnosable, benign, suspicious for malignancy, and malignancy. After the reclassification, the number of cases in each category changed significantly. The number of cases in the non-diagnostic and benign categories decreased from 21 to 9 and 629 to 547, respectively, and the reduced 94 cases were reassigned to the newer AUS, SFM, and MAL groups. In contrast, the malignant group had a 7-case increase, from 1060 to 1067. Additionally, 393 cases previously identified as “suspicious of malignancy” were redistributed to new doubtful categories (e.g., AUS and SFM), bringing the total to 480. The decrease in the number of cases in the ND group was attributable to stricter criteria in the ISRSFC. Because previous criteria were so extensive that fluid containing a small number of cells was classified as “undiagnosable” and cytopathologists frequently rely on their subjective judgment to make the diagnosis. According to the ISRSFC, some cases initially diagnosed as “undiagnosable” were reclassified as other groups. Detection of missed atypical cells in patients initially described as benign leads to the reclassification of AUS, SFM, and MAL.

In terms of ISRSFC, the proper amount of serous effusion sent for examination was not specified. Previous studies have recommended at least 50 to 75 mL.^[11,12]^ However, this hypothesis remains controversial. In our investigation, despite some samples being as small as 1 mL, there were still enough cells counted and shaped for further identification and classification. Therefore, the volume of material in the literature should be used as a suggestion and the final decision should be based on the actual situation.

In this study, there were 2103 cases, of which 9 (0.4%) were in the ND group, 547 (26%) in the NFM group, 94 (4.5%) in the AUS group, 386 (18.4%) in the SFM group, and 1067 (50.7%) in the MAL group. The proportion of ND was 0.4%, which is comparable to some reports^[3,10,13,14]^ but lower than other reports.^[9,15–17]^ The MAL category accounted for the majority of our samples, and the percentage of SFM cases was more significant than that in previous studies.^[9,18–21]^ This phenomenon stems from the fact that the source of serous effusion mainly comes from patients with tumors, and tumor conditions are the most common reason for fluid cytology. If a patient is diagnosed with SFM, physicians will consider it positive based on clinical symptoms and tumor history, thus depriving some SFM of the opportunity to be identified as MAL or NFM using adjunctive methods such as immunohistochemistry. In addition, if only a few highly atypical cells are present but the patient has a clear history of malignancy, SFM may be diagnosed. However, without a history of tumors, the same cells may not be sufficient to diagnose SFM, only AUS.

In our study, the ROM of pleural effusions were 50%, 31.0%, 37.0%, 91.8%, and 100% for ND, NFM, AUS, SFM, and MAL, respectively. The ROM for peritoneal effusion were 0, 21.0%, 36.8%, 85.2%, and 100% for ND, NFM, AUS, SFM, and MAL, respectively. The ROM of pericardial effusion were 100%, 8.0%, 36.4%, 100%, and 100% for the ND, NFM, AUS, SFM, and MAL, respectively. As can be seen, SFM has a more excellent ROM value. This result may be because our serous effusions were mainly from tumor patients, which may have produced a judgment bias. Furthermore, ROM values in the negative group were lower than those in the unsure group. This is consistent with the results of previous studies.^[9,17,21]^

The highest specificity and PPV were obtained when only M was considered positive, whereas the highest sensitivity was obtained when AUS, SFM, and M were considered positive. These findings are similar to those reported by Zhu et al.^[3]^ The highest diagnostic accuracy was observed when AUS and SFM were positive for pleural and peritoneal effusions, in agreement with Straccia et al,^[9]^ but unlike Lobo et al.^[22]^ The false-positive and false-negative diagnostic rates highly depend on the investigator’s experience. False-positive cases may be due to reactive atypia of the mesothelial hyperplasia. Conversely, false-negative results can be attributed to local invasion of the plasma membrane by malignant tumors or sampling errors.^[3]^

The most common malignancy in pleural and pericardial effusion is lung cancer, which is consistent with earlier results in the literature.^[3,18,22,23]^ Similar to the findings of Straccia et al,^[9]^ gastrointestinal malignancies were the most commonly diagnosed malignancies in the peritoneal effusions. Nevertheless, ovarian cancer is the most prevalent malignancy in peritoneal effusions, according to 2 recent studies.^[17,24]^

In the present study, a broad immunopanel was considered, including epithelial, mesothelial, lymphatic, and other markers. Positive immunohistochemical staining for CK7, thyroidtranscription factor-1, and Napsin A, for example, can identify lung adenocarcinoma, which is the most prevalent malignancy in pleural and pericardial effusions. Similarly, gastrointestinal cancer can be identified in the peritoneal effusion using specific stains for caudal type homeobox 2 and cytokeratin 20. Breast cancer is the most common cause of pleural effusion in women. Mammaglobin and GATA-binding protein 3 are immunohistochemical biomarkers used to identify the origin of breast cancer. estrogen receptor, progesterone receptor, and human epidermal growth factor receptor 2 are frequently used to predict response to clinical treatment. Serous carcinoma cells of the female reproductive system should be distinguished from the mesothelial cells. Although both mesothelial cells and serous epithelium express WT1, mesothelial cells usually lack other epithelial markers (e.g., CK7, cancer antigen 125, PAX8, and claudin 4) and express calretinin, which is often absent in epithelial cells.

This study was limited by 2 aspects. First, liquid-based cytology was not performed, and it could not be compared with conventional smears owing to the limited conditions of our department. Second, because most of our effusion samples came from patients with tumors, all groups had significant MAL, SFM, and ROM prevalence.

## 5. Conclusion

The ISRSFC is an easy-to-grasp reporting system that provides a consistent standard for better communication between physicians and pathologists. The system is also very sensitive for the detection of primary and secondary cancers. Furthermore, sufficient samples combined with auxiliary methods, such as immunohistochemistry, could further improve the accuracy of cytological diagnosis.

## Author contributions

**Data curation:** Haiping Yang.

**Formal analysis:** Jianyou Zhu.

**Methodology:** Jianyou Zhu.

**Supervision:** Pingjiang Wang.

**Writing – original draft:** Haiping Yang.

**Writing – review & editing:** Pingjiang Wang.

## References

[1] Michael CW. Serous fluid cytopathology: past, present, and future. Diagn Cytopathol. 2021;49:577–81. 33634959 10.1002/dc.24663

[2] Ebata T Okuma Y Nakahara Y. Retrospective analysis of unknown primary cancers with malignant pleural effusion at initial diagnosis. Thorac Cancer. 2016;7:39–43. 26816537 10.1111/1759-7714.12271 PMC4718124

[3] Zhu YL Ren WH Wang Q. A retrospective analysis of serous effusions based on the newly proposed international system for reporting serous fluid cytopathology: a report of 3633 cases in an oncological center. Diagn Pathol. 2022;17:56. 35780135 10.1186/s13000-022-01241-4 PMC9250735

[4] Davidson B. Molecular testing on serous effusions. Diagn Cytopathol. 2021;49:640–6. 32023012 10.1002/dc.24392

[5] Yu GH Glaser LJ Gustafson KS. Role of ancillary techniques in fluidcytology. Acta Cytol. 2020;64:52–62. 31018204 10.1159/000496568

[6] Pinto D Cruz E Branco D. Cytohistological correlation in pleural effusions based on the international system for reporting serous fluid cytopathology. Diagnostics (Basel). 2021;11:1126. 34203073 10.3390/diagnostics11061126 PMC8235437

[7] Chandra A Crothers B Kurtycz D. The International system for reporting serous fluid cytopathology. Acta Cytol. 2019;63:349–51. 31234178 10.1159/000501536

[8] Xu Y Hu AY Wang SM. A retrospective analysis of pleural effusion specimens based on the newly proposed International System for Reporting Serous Fluid Cytopathology. Diagn Cytopathol. 2021;49:997–1007. 34019334 10.1002/dc.24804

[9] Straccia P Chiappetta M Magnini D. Application of the International System for Reporting Serous Fluid Cytopathology (TIS): a retrospective institutional study. Cytopathology. 2022;33:305–11. 35213747 10.1111/cyt.13113

[10] Ahuja S Malviya A. Categorisation of serous effusions using the International System for Reporting Serous Fluid Cytopathology and assessment of risk of malignancy with diagnostic accuracy. Cytopathology. 2022;33:176–84. 34913541 10.1111/cyt.13089

[11] Abouzgheib W Bartter T Dagher H. A prospective study of the volume of pleural fluid required for accurate diagnosis of malignant pleural effusion. Chest. 2009;135:999–1001. 19017891 10.1378/chest.08-2002

[12] Rooper LM Ali SZ Olson MT. A minimum fluid volume of 75 mL is needed to ensure adequacy in a pleural effusion: a retrospective analysis of 2540 cases. Cancer Cytopathol. 2014;122:657–65. 25060164 10.1002/cncy.21452

[13] Farahani SJ Baloch Z. Are we ready to develop a tiered scheme for the effusion cytology? A comprehensive review and analysis of the literature. Diagn Cytopathol. 2019;47:1145–59. 31301215 10.1002/dc.24278

[14] Rodriguez EF Jones R Gabrielson M. Application of the International System for Reporting Serous Fluid Cytopathology (ISRSFC) on reporting pericardial effusion cytology. Acta Cytol. 2020;64:477–85. 32422631 10.1159/000507311

[15] Jha S Sethy M Adhya AK. Application of the International System for Reporting Serous Fluid Cytopathology in routine reporting of pleural effusion and assessment of the risk of malignancy. Diagn Cytopathol. 2021;49:1089–98. 34289263 10.1002/dc.24837

[16] Valerio E Nunes W Cardoso J. A 2-year retrospective study on pleural effusions: a cancer centre experience. Cytopathology. 2019;30:607–13. 31306514 10.1111/cyt.12755

[17] Kundu R Srinivasan R Dey P. Application of Indian Academy of Cytologists guidelines for reporting serous effusions: an institutional experience. J Cytol. 2021;38:1–7. 33935385 10.4103/JOC.JOC_224_20 PMC8078616

[18] Hou T Landon G Stewart J. The value of a tiered cytology diagnostic reporting system in assessing the risk of malignancy in indeterminate serous effusions. Cancer Cytopathol. 2021;129:75–82. 32809259 10.1002/cncy.22345

[19] Gokozan HN Harbhajanka A Lyden S. Root cause analysis of indeterminate diagnoses in serous fluids cytopathology. Diagn Cytopathol. 2021;49:633–9. 33125186 10.1002/dc.24653

[20] Rossi E Bizzarro T Martini M. The role of liquid based cytology and ancillary techniques in the peritoneal washing analysis: our institutional experience. PLoS One. 2017;12:e0168625. 28099523 10.1371/journal.pone.0168625 PMC5242474

[21] Sun T Wang M Wang H. Risk of malignancy assessment of the International System for Reporting Serous Fluid Cytopathology: experience in a community hospital setting and comparison with other studies. Cancer Cytopathol. 2022;130:964–73. 35994357 10.1002/cncy.22638

[22] Lobo C Costa J Petronilho S. Cytohistological correlation in serous effusions using the newly proposed International System for Reporting Serous Fluid Cytopathology: experience of an oncological center. Diagn Cytopathol. 2021;49:596–605. 32339444 10.1002/dc.24440

[23] Pergaris A Stefanou D Keramari P. Application of the International System for Reporting Serous Fluid Cytopathology with cytohistological correlation and risk of malignancy assessment. Diagnostics (Basel). 2021;11:2223. 34943460 10.3390/diagnostics11122223 PMC8700584

[24] Kolte S Zaheer S Aden D. Application of the international system for reporting serous fluid cytopathology on reporting various body fluids; experience of a tertiary care hospital. Cytojournal. 2022;19:52. 36128470 10.25259/Cytojournal_49_2021 PMC9479562

